# EXAMINING THE NON-LINEAR ASSOCIATION BETWEEN YEARS OF EDUCATION AND LATE-LIFE COGNITION

**DOI:** 10.1093/geroni/igad104.3320

**Published:** 2023-12-21

**Authors:** Sara Robinson, Jason Salemi, Hongdao Meng, Judith Rijnhart

**Affiliations:** University of South Florida, Tampa, Florida, United States; University of South Florida College of Public Health, Tampa, Florida, United States; University of South Florida, Tampa, Florida, United States; University of South Florida, Tampa, Florida, United States

## Abstract

Dementia, a rising health concern, is expected to impact 13 million adults in the United States by 2050. Low education is a well-established risk-factor for dementia. However, few studies have investigated possible non-linearity of the association between years of education and late-life cognition, nor have investigated gender differences in this association. This study aimed to investigate whether years of education is non-linearly associated with late-life cognition, and whether the size of this association differed across men and women. To address this aim, we used data from the U.S. representative Health and Retirement Study, encompassing a sample of 32,712 respondents with episodic memory data available between 1998 and 2018 and with complete data on self-reported education (median=12, interquartile range=12-15), gender (18,509 women, 14,203 men), and confounders (ethnicity, race, parental education, childhood immigration status, and birth year). Episodic memory was measured as immediate and delayed recall using a 10-word recall test. We used confounder-adjusted linear mixed-effects models to estimate the associations between years of education and immediate recall (model 1) and delayed recall (model 2). In both models, the estimated education-recall association was non-linear based on the statistically significant squared and cubic terms (p< 0.001). Generally, an increase in education was associated with an increase in immediate and delayed recall, and these associations were more pronounced for larger education values. There were no statistically significant gender differences in the education-recall associations in both models. These findings indicate the importance of assessing non-linearity of the association between years of education and late-life cognition.

